# Ball Magnets Clicked Together on the Epiglottis

**DOI:** 10.7759/cureus.8181

**Published:** 2020-05-18

**Authors:** Mark A Taylor, Stephen P Spanos, Stephen J Fenton, Katie W Russell

**Affiliations:** 1 Department of Surgery, University of Utah, Salt Lake City, USA; 2 Department of Anesthesiology, Primary Children's Hospital, Salt Lake City, USA

**Keywords:** neodymium, pharynx, foreign body, ingestion, magnet

## Abstract

Neodymium ball magnets are commonly ingested by children and are a risk of causing significant morbidity if not addressed appropriately. While most ingested magnets are located distal to the epiglottis in the gastrointestinal tract, they can rarely get lodged across tissues in the mouth and throat such as the epiglottis. Though rare, this represents an impending airway emergency and requires urgent treatment once identified. We present the case of a two-year-old, asymptomatic male who presented after ingesting two neodymium ball magnets that were found to be clicked together across his epiglottis, which were ultimately retrieved by bronchoscopy without complications.

## Introduction

Neodymium magnet ingestion is a persistent problem affecting both children and adolescents. The frequency of magnet ingestions increased significantly in 2002 due to the presence of these magnets in easily disassembled toys and household objects [1]. Cases in the US peaked at over 3000 in 2012 [1]. Due to this rapid increase in magnet ingestions, in 2011, the US Consumer Products Safety Commission began to enforce federal guidelines related to these magnets [1]. Subsequently, between 2012 and 2015, the number of estimated ingestions decreased by 13% per year [2]. Despite this and advocacy from physician and consumer groups, magnet ingestion continues to occur regularly [2].

We report a case of a two-year-old male who presented after ingesting two ball magnets that became attached on opposite sides of his epiglottis. The location of these magnets was unique because most magnets are located in the gastrointestinal tract distal to the epiglottis after ingestion. This case demonstrates the importance of looking throughout the gastrointestinal and respiratory tracts when magnets are ingested and illustrates the potential for respiratory distress if magnets are lodged in the airway.

## Case presentation

**Figure 1:**
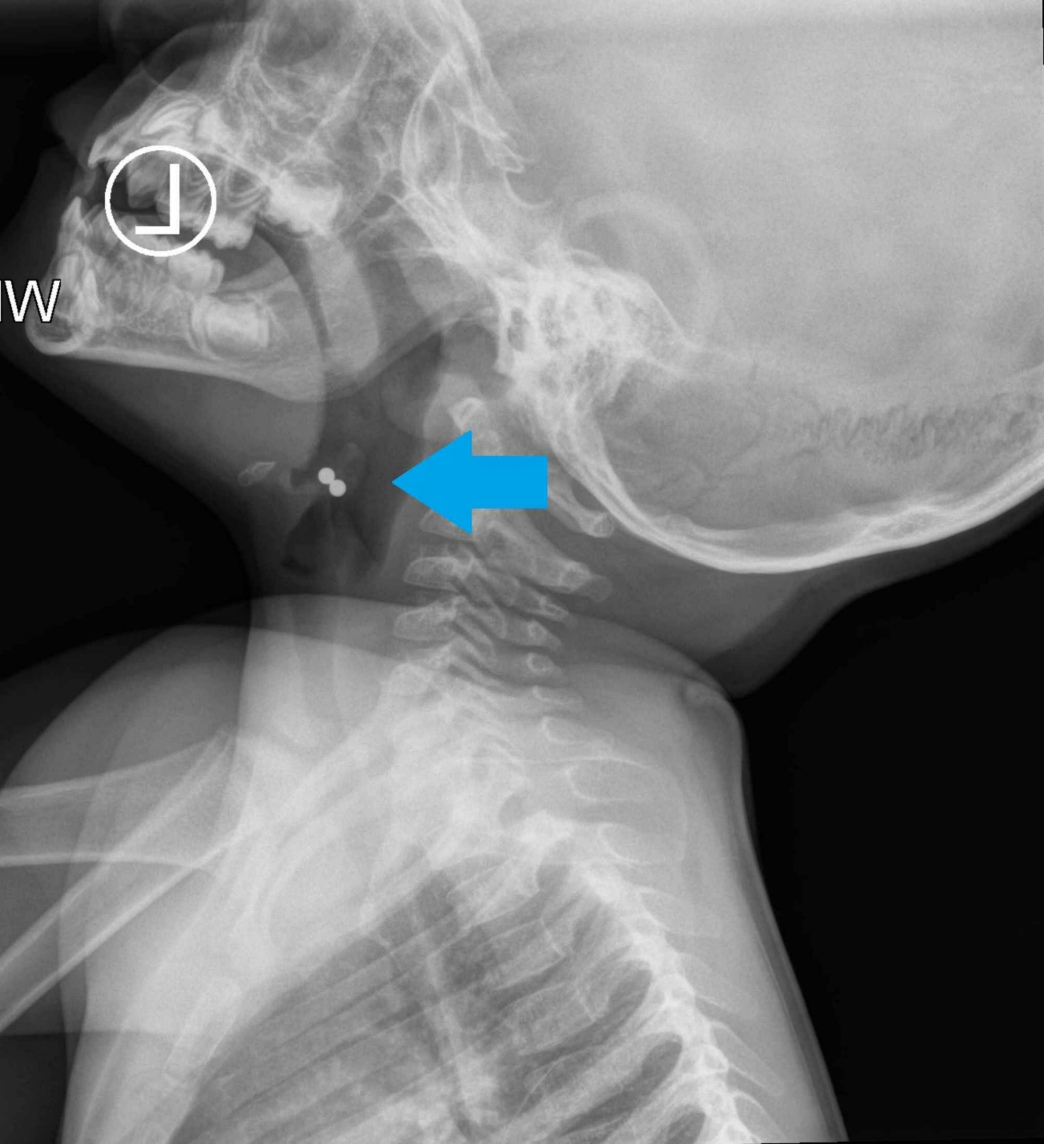
Plain film of the neck in a two-year-old that ingested neodymium ball magnets that are located at the level of the epiglottis

A two-year-old male presented to the emergency department after the suspected ingestion of two neodymium ball magnets earlier in the day. He was asymptomatic and did not experience respiratory distress to suggest the inhalation of the magnets or abdominal pain, abdominal distension, nausea, or vomiting to suggest the ingestion of the magnets. He had no significant medical or surgical history. On physical exam, he was breathing comfortably without stridor, wheezing, or drooling, and his abdominal exam was benign. A work-up for the ingestion of foreign bodies was performed, including X-rays of the head, neck, and abdomen, to locate the magnets, determine how many were ingested, and determine the relative location of the ball magnets to each other. On the X-ray of the neck, two ball magnets were noted to be situated around the epiglottis (Figure 1). All other plain films were negative for further ingested magnets.

**Figure 2:**
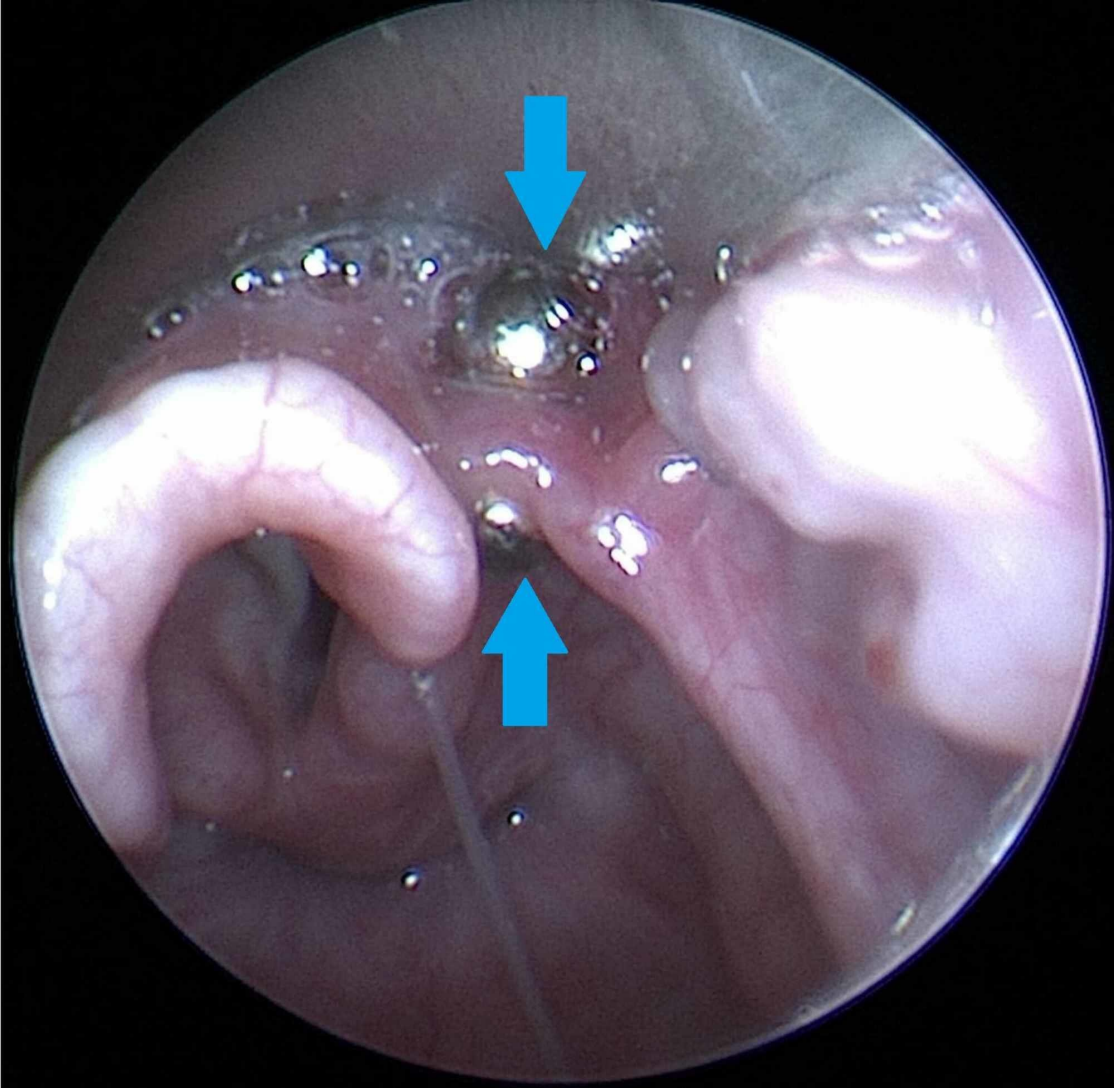
Bronchoscopic image of two neodymium ball magnets clicked together across the epiglottis in a two-year-old male

Because of the risk of impending respiratory distress, the patient was taken to the operating room for urgent bronchoscopy. After sedation in the operating room, a laryngoscope was initially used to locate the ball magnets, at which time they were noted to be clicked together across the epiglottis (Figure 2).

In order to maintain maximal control, a rigid bronchoscope and optical forceps were used to remove the magnets. The magnets were removed without issue (Video 1). After observing the patient in the recovery room to ensure no subsequent respiratory distress from the manipulation of the epiglottis, the patient was discharged home. On follow-up, the patient was doing well without any negative sequelae.

**Video 1:** Video of bronchoscopic retrieval of two neodymium ball magnets clicked together across the epiglottis in a two-year-old male

## Discussion

Neodymium ball magnets are significantly stronger than traditional ferrite magnets. They are often accessible to children because they are sold as desktop toys and are found in household objects such as refrigerator magnets [1]. Swallowing a single magnet is typically harmless, as it will pass through the gastrointestinal tract without attracting another magnet. However, if multiple magnets are swallowed independently, these magnets can attract each other across tissues. These magnets most commonly attached to each other across the loops of the bowel, creating pressure necrosis, which can lead to an enteroenteric fistula, bowel obstruction, and/or volvulus if not addressed [3]. The majority of patients that ingest magnets is initially asymptomatic. However, symptom progression varies depending on the number of magnets ingested and the location of the magnets when they become attracted to each other [4].

Given the sudden increase in neodymium magnet ingestion by children in the early 2000s when they were marketed as children’s toys, the North American Society for Pediatric Gastroenterology, Hepatology & Nutrition (NASPGHAN) produced guidelines for the management of these ingested magnets [5]. If a single magnet is identified on imaging, these guidelines suggest serial imaging to ensure the passage of the magnet [5]. If multiple magnets are identified, they recommend endoscopic or surgical intervention for most children due to the potential morbidity associated with ingestion [5]. If multiple magnets are clearly moving through the gastrointestinal tract together, there is a chance they will pass on their own. Magnets that fail to progress or that create obstructive symptoms are likely to require intervention.

While the majority of ingested magnets are located in the gastrointestinal tract distal to the epiglottis, this case is unique in that the magnets were noted to be clicked together on the lingual and laryngeal aspects of the epiglottis. While the patient was asymptomatic, this represents an airway urgency, as pressure necrosis of the epiglottis could lead to edema and respiratory distress or the magnets could be inhaled into the airway. It is important to recognize this as a rare, although, distinct location in which these magnets can become lodged. Examination of a patient with suspected magnet ingestion should include a physical and radiographic examination of the head, neck, and abdomen so that magnets in the mouth and throat are not missed. During the retrieval of these magnets, we believe it is important to remove the magnet on the laryngeal surface of the epiglottis first to prevent the accidental inhalation of the magnet upon removal.

## Conclusions

The ingestion of neodymium ball magnets is a relatively common source of morbidity in children. While they are most commonly located in the gastrointestinal tract distal to the epiglottis, they can rarely become clicked together across tissues in the mouth and throat. A thorough radiographic examination of the gastrointestinal and respiratory tracts, including the head and neck, should be performed in the work-up of a child that ingested magnets. Urgent removal is indicated if the magnets are located near the airway due to the risk of respiratory distress.

## References

[1] Reeves PT, Nylund CM, Krishnamurthy J, Noel RA, Abbas MI (2018). Trends of magnet ingestion in children, an ironic attraction. J Pediatr Gastroenterol Nutr.

[2] Kurowski JA, Kay M (2017). Caustic ingestions and foreign bodies ingestions in pediatric patients. Pediatr Clin North Am.

[3] Fenton SJ, Torgenson M, Holsti M, Black RE (2007). Magnetic attraction leading to a small bowel obstruction in a child. Pediatr Surg Int.

[4] Alfonzo MJ, Baum CR (2016). Magnetic foreign body ingestions. Pediatr Emerg Care.

[5] Kramer RE, Lerner DG, Lin T (2015). Management of ingested foreign bodies in children: a clinical report of the NASPGHAN Endoscopy Committee. J Pediatr Gastroenterol Nutr.

